# A Novel Encoder-Decoder Knowledge Graph Completion Model for Robot Brain

**DOI:** 10.3389/fnbot.2021.674428

**Published:** 2021-05-11

**Authors:** Yichen Song, Aiping Li, Hongkui Tu, Kai Chen, Chenchen Li

**Affiliations:** College of Computer, National University of Defence Technology, Changsha, China

**Keywords:** learning-based artificial intelligence, robot intelligence, human-robot interaction, knowledge graph reasoning and completion, knowledge graph embedding

## Abstract

With the rapid development of artificial intelligence, Cybernetics, and other High-tech subject technology, robots have been made and used in increasing fields. And studies on robots have attracted growing research interests from different communities. The knowledge graph can act as the brain of a robot and provide intelligence, to support the interaction between the robot and the human beings. Although the large-scale knowledge graphs contain a large amount of information, they are still incomplete compared with real-world knowledge. Most existing methods for knowledge graph completion focus on entity representation learning. However, the importance of relation representation learning is ignored, as well as the cross-interaction between entities and relations. In this paper, we propose an encoder-decoder model which embeds the interaction between entities and relations, and adds a gate mechanism to control the attention mechanism. Experimental results show that our method achieves better link prediction performance than state-of-the-art embedding models on two benchmark datasets, WN18RR and FB15k-237.

## 1. Introduction

With the development of science and technology, significant progress has been achieved in robotics that the types and application fields of robots are constantly enriched. These robots have played key roles in reducing tedious work. They provide optimal user service and improve the convenience of life. The popularity of various kinds of robots is an inevitable trend.

The emergence of learning intelligent social robots means that robots have truly begun to play roles in people's daily lives, such as pepper and buddy. There are some typical applications, such as greeting conversations, question responses, interest recommendations, and risk management (Gu et al., 2021). Huge information is need at the backend of these services. However, the traditional search engine will be affected by the combination of information, resulting in an increase in search volume and a decrease in accuracy. Knowledge with unique meaning and with the goal of solving practical problems can avoid this problem well.

The “Robot Brian” is taken the same as the brain for humans, which stores and infers knowledge to support other behaviors. Knowledge base is usually used to work as the brain of an intelligent robot. Unlike general applications that implicitly encode information in programs, it can explicitly express the corresponding knowledge of actual problems. Providing continuous knowledge support for robots through the knowledge base is equivalent to injecting “thought” into the robots to realize real intelligence true intelligence. Knowledge base construction is a core configuration for intelligent robots. Without a knowledge base, a robot cannot answer any questions. The richer the knowledge base, the more intelligent the robot will have when interacting with users.

A large amount of research work in knowledge representing, web data mining, natural language processing and other fields are dedicated to acquiring large-scale knowledge (Jia et al., 2021), providing rich knowledge bases for building the intelligent brain of robots. In order to facilitate computer processing and understanding, we express the knowledge base in a more formal and concise way, that is, a highly structured knowledge graph composed of triples (*e*_*h*_, *r*_*k*_, *e*_*t*_). The Knowledge Graphs (KG) not only provides robots with a more human-like representation of the world, but also provides a better way to organize, manage and utilize massive amounts of information.

Although the large-scale knowledge graphs already contain a large amount of entity and relation information, they are still incomplete compared with existing knowledge and newly added knowledge (Zhao et al., 2020). Through knowledge graphs and knowledge self-learning, problems in the knowledge system can be found, and knowledge can be supplemented and enhanced so that the robot's knowledge base can be continuously improved and evolved. There is no end to the optimization and completion of the knowledge base, just as there is no end to human learning, this is research work that needs continuous improvement and development.

In order to alleviate the above problems, researchers have proposed a knowledge graph embedding method, which predicts missing links based on existing facts so as to expand the knowledge base. Its purpose is to learn low-dimensional vector representations of all entities and relationships, so as to simplify operations while the original structured information of the knowledge graph is retained. These knowledge graph embedding methods are widely divided into translation models (Bordes et al., 2013; Ji et al., 2015; Lin et al., 2015), semantic matching model (Nickel et al., 2011, 2016; Yang et al., 2015; Trouillon et al., 2016), and neural network models (Dettmers et al., 2018; Shang et al., 2019; Vashishth et al., 2020). The related work will be introduced in detail in section 2.

Compared with neural network models, the other types of models are all shallow models, which leads to problems with poor expressiveness. Therefore, more and more complex and deeper models, which have better expressive performance and have achieved competitive success in modeling knowledge graphs, have been proposed in recent years. But these existing models, such as Dettmers et al. (2018), Nguyen et al. (2018), Shang et al. (2019), and Vashishth et al. (2020), are more focused on entity representation learning, and the importance of relation representation learning are ignored, let alone the cross-interaction between entities and relations. The interaction between entities and relations plays an important role in knowledge graph representation learning that the entities and relations in the knowledge graph will influence each other and influence the prediction of new triples as they do in the real world.

In this paper, a method of knowledge reasoning and completion based on neural networks on the knowledge graph is designed for robots to simulate the reaction and learning process of human brains. Our model adopts the encoder-decoder model. The encoder model improved the KBGAT model with a gate mechanism to control the attention mechanism and use entity embeddings to update relation embeddings. The decoder model uses Conv-TransE and Conv-TransR to achieve state-of-the-art efforts. This method can enable the robot to quickly search for information, predict answers, and complete knowledge from the knowledge base, to better understand user intent and interact with users more intelligently.

## 2. Related Work

In this section, we mainly introduce the work related to our Large-scale Knowledge Graph reasoning and completion methods for robots. As one of the research hotspots, Large-scale Knowledge Graph reasoning and completion has attracted extensive attention from academia and industry. Thus, many different types of methods are born, such as the translation model, the bilinear model, the hyperbolic geometry model, the neural network model, the rotate model, and so on. Among these different kinds of methods, the knowledge graph Embedding method is the closest to human expression, which can be regarded as languages for computers and machines like robots. The knowledge graph Embedding method generally includes the following types of models: (i) translation models; (ii) models based on semantic matching; (iii) models based on neural networks; (iv) models with additional information. We will mainly introduce the work related to translation models and neural network based models related to our work in the following.

The translation model represented by TransE (Bordes et al., 2013) uses a simple vector form to represent the entities and relations in the knowledge graph. TransE (Bordes et al., 2013) regards relation as the conversion from the head entity *e*_*h*_ to the tail entity *e*_*t*_, and uses *e*_*h*_+*r*_*k*_ = *e*_*t*_ to determine whether the given triplet is correct. In order to make up for the defect that TransE can only handle the 1–1 relation, TransH (Wang et al., 2014), TransD (Ji et al., 2015), TransR (Lin et al., 2015), and other models have increased the ability to handle multiple relations and semantics and enhanced the knowledge embedding model. It shows that entities and relations can also be embedded in other spaces besides real number space. TransG (Xiao et al., 2015) introduces Gaussian distribution to solve the problem of multi-relational semantics to capture the uncertainty of entities and relations. TorusE (Ebisu and Ichise, 2018) is the first model to embed objects outside the space of real or complex numbers and select a torus (compact Lie group) as the embedding space.

Neural network-based embedding models have received extensive attention in recent years. These methods include embedding models based on convolutional neural networks (CNN) and graph convolution networks (GCN). For example, convE (Dettmers et al., 2018), ConvKB (Nguyen et al., 2018), and InteractE (Vashishth et al., 2020) are both relational prediction models based on convolutional neural networks. ConvE (Dettmers et al., 2018) stacks the embeddings of head entity and relation into a 2-dimensional matrix, and performs convolution operation to extract features with fewer parameters and faster calculations. IntercatE (Vashishth et al., 2020) increases the expressive power of ConvE and expands the interaction between entities and relations through three key ideas—feature permutation, feature reshaping, and circular convolution. ConvKB (Nguyen et al., 2018) represents each triple as a 3-column matrix where each column vector represents a triple element and feeds this matrix to a 1D convolution layer to generalize transitional characteristics in transition-based embedding models.

Graph convolution networks have made great progress in improving the efficiency of node representation in the graph, and it is also applied in the knowledge graph by researchers. Graph convolution network (GCN) (Kipf and Welling, 2017) gathers information for node(entity) from its neighbors with equal importance. Velickovic et al. (2018) introduce a graph attention network (GAT) to learn to assign varying levels of importance to node(entity) in every neighbor. However, these models are unsuitable for KGs, since they ignore that edges (triples) play different roles depending on the relation they are associated with in KGs. SACN (Shang et al., 2019) extends the classic GCN to a weighted graph convolutional network (WGCN) as an encoder, and uses a convolution model Conv-TransE as a decoder to construct an end-to-end model. WGCN weighs the different types of relations differently when aggregating multiple single-relation graphs into a multi-relation graph and the weights are adaptively learned during the training of the network. But WGCN inherits GCN's shortcomings in that it treats the same relation type for different entities of the same weight. Nathani et al. (2019) extends classic GAT to KBGAT by incorporating relation and neighboring node features in the attention mechanism and uses KBGAT as encoder and ConvKB (Nguyen et al., 2018) as a decoder.

The above-mentioned models have achieved good performance in knowledge graph embedding for knowledge graph reasoning and completion. However, as far as we know, few works consider the cross-interaction of entities and relations when designing models. Our proposed model uses a variant of the graph attention network (GAT) as the encoder and uses variants of ConvE [Conv-TransE (Shang et al., 2019), Conv-TransR] as decoder, to achieve the simultaneous capture of entity-to-relation and relation-to-entityc̱ross-interaction.

## 3. Model

**Figure 1:**
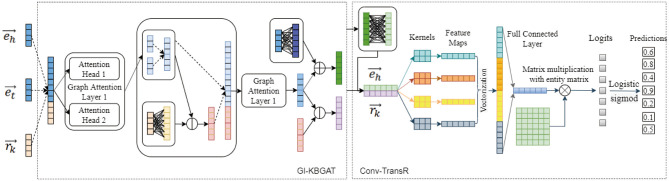
Model Architecture. The encoder in the left uses is our GI-KBGAT to obtain the embeddings of entity and relation. The right decoder model feeds the embeddings of *e*_*h*_ and *r*_*k*_ and use logits activation function to calculate scores. Dashed arrows in the figure represent concatenation operation. The dimension of vector $\vec{e_{h}}$ and $\vec{r_{k}}$ in the middle is the same with the left in fact. We here use 4 dimension to simply the representation of the model.

This section begins by introducing some notations and definitions used in the rest of this article. This is followed by an introduction of our encoder model GI-KBGAT, an improved Graph Attention Network for KG, which considers gate mechanism on multi-head attention and interaction between entities and relations to generate embeddings. Finally, we describe our decoder network based on Conv-TransE (Conv-TransR). The architecture of our model is as shown in Figure 1.

### 3.1. Notations and Definitions

The knowledge graph is defined as G=(E,R,T), where $E=\left\{e_{1},e_{2},...e_{N}\right\}$ and $R=\left\{r_{1},r_{2},...r_{K}\right\}$ represent the set of entities (nodes) and relations. *N* is number of entities and *K* is number of relations. T denotes the triples (edges) of the form $t_{htk}=\left(e_{h},r_{k},e_{t}\right)\in E\times R\times E$, where *e*_*h*_ is head entity, *e*_*t*_ is tail entity, and *r*_*k*_ is the relation between head and tail entity. In particular, entity *e*_*h*_ and *e*_*t*_ in this paper refer to the head entity and the tail entity, respectively, while other entities with subscripts, such as entity *e*_*i*_ are not specified. Table 1 explains the notations that will be used in the rest of this article.

**Table 1:** Some of the notations and explanations used in this paper.

**Notation**	**Explanation**	**Notation**	**Explanation**
**E, R**	Embedding matrices for entities and relations	[∥]	Concatenation of vectors
$\vec{e_{i}},\vec{r_{k}}$	Embedding vectors of entity *e*_*i*_ and relation *r*_*k*_	∣S∣	Number of elements in set S
$N_{i}$	Neighbor sets of entity *e*_*i*_	ϕ	LeakyReLU function
$R_{ij}$	Relation sets connecting entities *e*_*i*_ and *e*_*j*_	σ	Activate function
$P_{k}$	Entity pair (*e*_*h*_, *e*_*t*_) sets with relation *r*_*k*_	ψ	ReLU function

### 3.2. Encoder: GI-KBGAT

As shown in section 2, most existing models ignore the cross-interaction of entities and relations. They only use relations to updating entities, but ignore the effects of entities on relations. We improve the KBGAT (Nathani et al., 2019) by modifying the update process of embeddings of entities and relations to consider the interaction between entities and relations in the update process, and adding a gate mechanism to the attention mechanism for control.

Our model uses the initial embeddings of entities and relations as input, and the following layers use the embeddings obtained from its previous layer as input. The same as Nathani et al. (2019)'s GAT model, in order to learn the embeddings of entity *e*_*i*_, we aggregate features of triples associated with it. The triple's embedding is learned by performing a linear transformation over the concatenation of entity and relation vectors corresponding to it, as shown in Equation (1).

(1) $$\begin{array}{r} \vec{t_{htk}}={\text{W}}_{t}\left[\vec{e_{h}}∥\vec{e_{t}}∥\vec{r_{k}}\right] \end{array}$$

Where $\vec{t_{htk}},\vec{e_{h}},\vec{e_{t}}$, and $\vec{r_{k}}$ represent the embeddings of triple *t*_*htk*_, entity *e*_*h*_, *e*_*t*_, and relation *r*_*k*_, respectively, **W**_*t*_ denotes the linear transformation matrix. To measure the importance of each triple *t*_*htk*_ for entity *e*_*h*_, the LeakyRelu non-linearity activation function ϕ is used to get the absolute attention value, the activate vector is defined as $\vec{b}$, and the softmax function is used to obtain the relative attention values α_*htk*_, as shown in Equation (2).

(2) $$\begin{array}{r} \alpha_{htk}=\frac{exp\left(\phi \left(\vec{b}\vec{t_{htk}}\right)\right)}{\sum_{n\in N_{h}}\sum_{r\in R_{hn}}exp\left(\phi \left(\vec{b}\vec{t_{hnr}}\right)\right)} \end{array}$$

Then, the new embedding of entity *e*_*h*_ is obtained by aggregating the features of the triples associated with *e*_*h*_ through weighted by their attention values. As shown in Equation (3), attention values are used to calculate the linear combination of triples(neighbor) features, and the embedding is obtained with a activate function σ.

(3) $$\begin{array}{r} \vec{e_{h}}=\sigma \left(\underset{t\in N_{h}}{\sum }\underset{k\in R_{ht}}{\sum }\alpha_{htk}\vec{t_{htk}}\right) \end{array}$$

To stabilize the learning process and encapsulate more information, our encoder also uses a gated multi-head attention mechanism inspired by Vaswani et al. (2017), Velickovic et al. (2018), and Zhang et al. (2018). Considering *M* independent attention heads, *M* embeddings for an entity are obtained. For example, the embedding of entity *e*_*h*_ calculated by the *m*−*th* attention head is represented as ${\vec{e_{h}}}^{m}$. These embeddings of an entity are concatenated with independent gate value $g_{h}^{m}$ except the last layer (for which we use the mean pooling). The final entity embedding update equation is as follows:

(4) $$\begin{array}{r} \vec{e_{h}}=\sigma \left(\left[{∥}_{m=1}^{M}\left(g_{h}^{m}\cdot \sigma {\left(\underset{t\in N_{h}}{\sum }\underset{k\in R_{ht}}{\sum }\alpha_{htk}\vec{t_{htk}}\right)}^{m}\right)\right]\right) \end{array}$$

For the relation update, we propose an update mechanism that uses the same projection operation as the TransR model (Lin et al., 2015). Similar to GAT, TransR model holds that an entity is a complex of various attributes, and different relations focus on different attributes of the entity. TransR uses the projection matrix **M_r_** to project the head entity *e*_*h*_ and tail entity *e*_*t*_ into the corresponding relation space, and defines the score function as $f_{r}(e_{h},e_{t})=∥\vec{e_{h}}M_{r}+\vec{r}-\vec{e_{t}}M_{r}{∥}_{2}^{2}$. Inspired by TransR, we project the head entity *e*_*h*_ and the tail entity *e*_*t*_ of a triple into the relation space with a projection matrix **W**_*r*_, and update their relation *r*_*k*_ as Equation (5).

(5) $$\begin{array}{r} \vec{r_{k}}=\frac{1}{|P_{k}|}\underset{\left(h,t\right)\in P_{k}}{\sum }\left(\vec{e_{t}}-\vec{e_{h}}\right){\text{W}}_{r} \end{array}$$

In order not to lose the initial embeddings information during training, our model design a gated mechanism to aggregate the initial embeddings and the updated embeddings with learnable gate values. The equation is as shown in Equation (6).

(6) $$\begin{array}{r} \begin{array}{cc} \text{E} & =g_{ei}{\text{E}}_{initial}{\text{W}}_{te}+g_{eu}{\text{E}}_{update}\\ \text{R} & =g_{ri}{\text{R}}_{initial}{\text{W}}_{tr}+g_{ru}{\text{R}}_{update} \end{array} \end{array}$$

Where **W**_*te*_ and **W**_*tr*_ are the linearly transform matrices for the initial embeddings of entity **E**_*initial*_ and relation **R**_*initial*_, *g*_*ei*_, and *g*_*ri*_ are the memory gate for the initial embeddings of entity **E**_*initial*_ and relation **R**_*initial*_, *g*_*eu*_, and *g*_*ru*_ are the update gate for the updated embeddings of entity **E**_*update*_ and relation **R**_*update*_ obtained by Equations (4) and (5), respectively. The scoring function for the GI-KBGAT method is defined as follows:

(7) $$\begin{array}{r} f\left(\Omega \right)=\underset{t_{htk}\in S}{\sum }\underset{t_{htk}^{\prime }\in S^{\prime }}{\sum }\left(∥\vec{e_{h}}+\vec{r_{k}}-\vec{e_{t}}{∥}_{1}-∥\vec{e_{h}^{\prime }}+\vec{r_{k}^{\prime }}-\vec{e_{t}^{\prime }}{∥}_{1}\right) \end{array}$$

Where S and $S^{\prime }$ denotes the set of valid triples [*t*_*htk*_ = (*e*_*h*_, *r*_*k*_, *e*_*t*_)] and invalid triples [$t_{htk}^{\prime }=\left(e_{h}^{\prime },r_{k}^{\prime },e_{t}^{\prime }\right)$], respectively, ∥·∥_1_ means L1-norm dissimilarity.

### 3.3. Decoder

The convolutional structure is used as the base model of our decoder, which transforms the embedding vector to another space and possesses powerful feature extraction ability and good parameter efficiency. The decoder takes the embeddings of entity and relation trained from the encoder as input. We test both Conv-TransE (Shang et al., 2019), and Conv-TransR, which keeps the translational property of TransR ($\vec{e_{h}}\text{W}+\vec{r_{k}}\approx \vec{e_{t}}\text{W}$) with 1D convolution inspired by Conv-TransE to be consistent with encoder, as decoder as shown in Figure 1.

The only difference between Conv-TransR and Conv-TransE is that the Conv-TransR model has one more project matrix for entities than Conv-TransE. Following shows the model of Conv-TransR: Conv-TransR uses matrix **W** to project the entity *e*_*h*_ into its corresponding relation *r*_*k*_'s space, the result is $\vec{e_{h}}\text{W}$. This result is then stacked with its corresponding relation embedding $\vec{r_{k}}$ to get $\left[\vec{e_{h}}\text{W},\vec{r_{k}}\right]$ as the input of convolutional network. The convolutional network uses different filters(kernels) ω∈ℝ^2 × *F*^(*F*∈{1, 2, 3...}) to generate different feature maps as Conv-TransE. The scoring function for the Conv-TransR method is defined as below:

(8) $$\begin{array}{r} g\left(e_{h},r_{k},e_{t}\right)=\tau \left(\psi \left(\left[∥\psi \left(\left[\vec{e_{h}}\text{W},\vec{r_{k}}\right]⊛\omega \right)\right]{\text{W}}_{c}\right)\vec{e_{t}}\right) \end{array}$$

Where ⊛ represents a 1D convolution operation, [∥] denotes vector concatenation which concatenates features output from convolution with different filters ω, **W**_*c*_ is a learnable weight matrix for linear transformation to projected the concatenation embedding into the tail entity *e*_*t*_ space, ψ is chosen to be a ReLU non-linear function, then the calculated embedding is matched to tail entity *e*_*t*_ by an appropriate distance metric, and the logistic sigmoid function τ is used for scoring finally.

## 4. Experiments and Results

### 4.1. Datasets

Through continuous learning, the data scale of intelligent robots will only increase. Therefore, when evaluating our proposed method, we ignore the small datasets and chose two large datasets WN18RR (Dettmers et al., 2018) and FB15k-237 (Toutanova et al., 2015) as the benchmark datasets. WN18RR and FB15k-237 are improved versions of two common datasets WN18 and FB15k (Bordes et al., 2013) derived from WordNet and freebase, respectively, in which all inverse relations have been deleted to prevent direct inference of test triples by reversing training triples. Table 2 provides statistics of them.

**Table 2:** Statistics of the experimental datasets.

**Dataset**	**# Entities** **∣E∣**	**# Relations** **∣R∣**	**# Edges** ∣T∣				**Mean** in-degree	**Median** in-degree
			**Training**	**Validation**	**Testing**	**Total**		
WN18RR	40,943	11	86,835	3,034	3,134	93,003	2.12	1
FB15k-237	14,541	237	272,115	17,535	20,466	310,116	18.71	8

### 4.2. Training Settings

We follow a two-step training procedure that, we first train our GI-KBGAT to encode information about the graph entities and relations and then train decoder model Conv-TransR to perform the link prediction task. For encoder training, we use the margin ranking loss, use Adam to optimize all the parameters with the initial learning rate set at 0.001, set the entity and relation embedding dimension of the last layer to 200, and set the other hyper-parameters for each dataset to be the same as KBGAT (Nathani et al., 2019). For decoder training, we use the standard binary cross-entropy loss with label smoothing, set the size and number of the kernel to 9 and 200, respectively, and set the other hyper-parameters for each dataset to be the same as InteractE (Vashishth et al., 2020).

### 4.3. Evaluation Protocol

Following the previous work, we use the filtered setting (Bordes et al., 2013) that all valid triples are filtered out from the candidate set while evaluating test triples. The performance is reported on the standard evaluation metrics: Mean Reciprocal Rank (MRR) and the proportion of correct entities ranked in the top 1, 3, and 10 (Hits@1, Hits@3, Hits@10).

### 4.4. Results and Analysis

Table 3 presents the experimental results of our methods and several baseline methods on FB15K-237 and WN18RR test sets. In which all values are presented in percentage. Among these baseline methods, the methods in the first box, namely TransE (Bordes et al., 2013), ConvE (Dettmers et al., 2018), ConvKB (Nguyen et al., 2018), Conv-TransE (Shang et al., 2019), and InteractE (Vashishth et al., 2020), have their results taken from the original paper and can be resumed to acceptable results. We compared our methods with these methods in Table 3 to label the best score in bold.

**Table 3:** Experimental results on FB15K-237 and WN18RR test sets.

**Models**	**FB15k-237**				**WN18RR**			
	**MRR**	**H@1**	**H@3**	**H@10**	**MRR**	**H@1**	**H@3**	**H@10**
TransE Bordes et al., 2013	29.4	–	–	46.5	22.6	–	–	50.1
ConvE Dettmers et al., 2018	32.5	23.7	35.6	50.1	43	40	44	52
ConvKB Nguyen et al., 2018	**39.6**	–	–	51.7	24.8	–	–	52.5
Conv-TransE Shang et al., 2019	33	24	37	51	46	43	47	52
InteractE Vashishth et al., 2020	35.4	**26.3**	–	53.5	46.3	43.0	–	52.8
SACN Shang et al., 2019	35	26	39	54	47	43	48	54
KBGAT Nathani et al., 2019	20.5	11.4	22.8	39.6	40.4	32.2	44.8	55.4
Our encoder model (+ ConvTransE)	35.5	**26.3**	**39.1**	**53.8**	45.9	42.8	46.7	52.5
Our encoder model (+ ConvTransR)	33.9	24.4	37.6	52.8	**46.6**	**43.4**	**47.9**	**52.9**
Our encoder model (+ InteractE)	35.5	26.2	**39.2**	**54.1**	**46.7**	**43.5**	**48.1**	**52.9**

*All values are in percentage and the best scores of our model is in bold regardless of the second box (SACN and KBGAT)*.

Since our method is inspired by methods SACN (Shang et al., 2019) and KBGAT (Nathani et al., 2019) that we present their results in the second box. The results of the SACN model are obtained from its corresponding paper, but this model requires a large GPU to train and these results can not be reproduced with the authors' code. KBGAT has test data leakage in its original implementation that the results in its paper are not credible. In our experiment results table, we fix the problem and show the correct results of the model.

We first compare our model use Conv-TransE as the decoder with the Conv-TransE model. Our model performs better than Conv-TransE on both datasets. Especially in the FB15K-237 dataset, our model improves upon Conv-TransE's MRR by a margin of 7.6%, Hits@1 of 9.6%, Hits@3 of 5.7%, and Hits@10 of 5.5%. In the WN18RR dataset, our model improves upon Conv-TransE's Hits@10 by a margin of 1.0%. Under the same accuracy, our model achieves the same performance on the other metrics compared with Conv-TransE.

Second, we compare our model use InteractE as the decoder with the InteractE model to better prove the effectiveness of our encoder. As shown in the Table 3, compared with the original model, most metrics of InteractE have been improved after our encoder model is added. For example, our model with InteractE improves upon InteractE's MRR by a margin of 0.3% and Hits@10 of 1.1% in the FB15K-237 dataset.

Third, we compare our model with the other baseline models. In the FB15K-237 dataset, our model with Conv-TransE as decoder achieves the best performance in Hit@3 and Hit@10, and tied for the best in Hit@1. In the WN18RR dataset, our model with Conv-TransR as decoder achieves the best performance in all metrics. Meanwhile, these two models both can achieve the top three effects on the other datasets. In conclusion, our model can achieve the best results on both datasets FB15K-237 and WN18RR.

**Figure 2:**
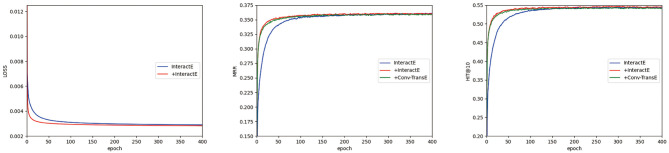
The convergence study of InteractE and our encoder model with InteractE as decoder (represented by “+InteractE”) and with Conv-TransE as decoder (represented by “+Conv-TransE”) in FB15k-237 using the validation set. Here we only report the results of loss, MRR and Hit@10.

Figure 2 shows the convergence of the three models: InteractE, our encoder model with InteractE as the decoder, and Conv-TransE as the decoder. Because Conv-TransE uses a different loss function, we do not put its loss result for comparison. We can see that our models (the red line and green line) are always better than InteractE (the blue line) under MRR and Hit@10. And our models converge faster than InteractE.

### 4.5. Ablation Experiment

In order to prove the validity of our model, we do some ablation experiments on WN18RR dataset to show the influences of different parts of our model. The results of the ablation experiments are shown in Table 4. Compared with the KBGAT model, our improved encoder with the KBGAT decoder (convKB) can achieve better performance as shown in the first box. After changing the decoder methods, our method performs more superior as in the second box. Combined with Table 3, the decoders we use are both better than the ConvKB which is used by KBGAT, which shows the effectiveness of our decoder chosen.

**Table 4:** Ablation experimental results on WN18RR test sets.

**Models**	**WN18RR**			
	**MRR**	**H@1**	**H@3**	**H@10**
KBGAT encoder (+ KBGAT decoder)	40.4	32.2	44.8	55.4
Our encoder model (+ KBGAT decoder)	41.1 (+0.7)	33.1 (+0.9)	45.5 (+0.7)	55.9 (+0.5)
Our encoder model (+ ConvTransR)	46.6	43.4	47.9	52.9
−gate	46.1 (−0.5)	43.0 (−0.4)	47.3 (−0.6)	52.4 (−0.5)
−rel	46.2 (−0.4)	43.0 (−0.4)	47.5 (−0.4)	52.7 (−0.2)
−gate − rel	46.1 (−0.5)	42.9 (−0.5)	47.3 (−0.6)	52.2 (−0.7)

To better show the influences of our innovation in KBGAT, we also test the influence of different parts in our encoder. We separately remove the gate mechanism, the interaction mechanism, and both of them to see the impact on results. The results are shown in the second box of Table 4. The gate mechanism and the interaction mechanism both perform a similar influence on the encoder model. And the best result can be achieved by combining them in the KBGAT model as the encoder model.

For these results, we conclude that our encoder-decoder model can better the expressive performance of entity embeddings and relation embeddings, and can achieve competitive success in modeling knowledge graphs. Since the hyper-parameters of our model for each dataset are set to the same as the existing methods and no parameter tuning is performed to obtain the best performance, we believe that the performance of our model can still be improved with parameter tuning.

## 5. Conclusion

In this paper, we propose a novel approach for knowledge graph relation prediction, which can be used in intent understanding in human-robot interaction and in robots' knowledge graph completion. Our methods can work well in large-scale knowledge graphs and can be extended to learn embeddings for various applications of robots, such as dialog generation and question answering.

In the future, we intend to extend our method to work as an end-to-end work and consider the attribute information and temporal information into our model to improve the ability to handle complex knowledge graphs. And we also intend to test our work in a real robot's “brain” to test the ability of our model in actual work.

## Data Availability Statement

Publicly available datasets were analyzed in this study. This data can be found at: two datasets used: WN18RR and FB15k-237. Download from https://github.com/TimDettmers/ConvE.

## Author Contributions

YS, KC, and CL contributed to the conception and design of the study. KC wrote the sections of the code. CL wrote the parts of the first draft of the manuscript. YS organized the experiments and the manuscript. AL and HT directed the whole work and revised the manuscript. All authors approved the submitted version.

## Conflict of Interest

The authors declare that the research was conducted in the absence of any commercial or financial relationships that could be construed as a potential conflict of interest.
